# Exploring genetic counselors' interest and role in transitional care discussions for pediatric patients with neurodevelopmental conditions

**DOI:** 10.1002/jgc4.1992

**Published:** 2024-11-14

**Authors:** Molly Lombard, Leah Wetherill, Jennifer Ivanovich, Deborah Hamby, Trisha Neidlinger

**Affiliations:** ^1^ Department of Medical and Molecular Genetics Indiana University School of Medicine Indianapolis Indiana USA; ^2^ Indiana University Health Physicians Indianapolis Indiana USA; ^3^ Department of Pediatrics Indiana University School of Medicine Indianapolis Indiana USA

**Keywords:** adolescents, genetic counseling, genetic counselors, neurodevelopmental conditions, transitional care

## Abstract

Previous studies have examined the perspectives of healthcare providers regarding their role in the transition process of pediatric patients with neurodevelopmental conditions (NDCs), but the perspective of a genetic counselor has yet to be considered. This study explored genetic counselors' current practices and attitudes regarding care for pediatric patients with NDCs as they transition into adult care. Genetic counselors (GCs) currently seeing patients with NDCs were recruited through a cross‐sectional online survey. Questions included demographics, current practices with patients aged 0–15 years vs. patients 16–22 years, self‐confidence, attitudes, and barriers regarding a genetic counselor's role in medical transition. A total of 51 surveys were included in the analysis. The majority (76.7%) of GCs were interested in playing a role in the transition process. Furthermore, all participants perceived transition planning for pediatric genetic patients with NDCs as somewhat important or important. Most GCs (70.0%) discussed topics of transition with patients over 18 years of age. These results demonstrate GCs' interest in assisting patients and families with the transition of individuals. Incorporating a genetic counselor skill set into transition discussions with patients with NDCs could improve the transition process and help to meet the standards of care called for by the American Academy of Pediatrics.

**What is known about this topic**

The American Academy of Pediatrics (AAP) defines standards of care that should be met for children transitioning to adulthood. The AAP 2018 report stated transition care needs for youth with and without special healthcare needs were not being met.

**What this paper adds to this topic**

This study explores GCs' current practices and attitudes toward discussing transitional care with their pediatric patients with neurodevelopmental conditions. The majority of GCs were interested in contributing their skills toward improving the transition into adult medical care during post‐test counseling appointments.

## 1 INTRODUCTION

Neurodevelopmental conditions (NDCs) are characterized by impairment in the areas of behavior, language, learning, or physical ability (Du et al., 2022; Savatt & Myers, 2021). NDCs include attention deficit and hyperactivity disorder, general learning disability, and autism and are present in ~17% of children ages 3–17 years of age (Zablotsky et al., 2019).

Transitional care can be defined as the period in which medical, psychological, and social care are pivoted from a pediatric to an adult perspective (Berens et al., 2020). Children with NDCs require additional care throughout their lifetime, thus the transition process should address concerns such as daily care, as well as legal and financial guardianship/conservatorship (Culnane et al., 2021). In 2018, the American Academy of Pediatrics (AAP) reported 84% of youth with special healthcare needs (SHCN) are not provided the national healthcare standards for transition (White & Cooley, 2018). The standards not being met focus on adolescent patients having the opportunity to speak on and plan for their future adult care with providers. At this transition age, youth should be encouraged to understand their diagnosis and how to best advocate for their health needs. Genetic counselors are positioned to aid in breaking down a complex genetic diagnosis for a young adult that may have only been discussed with their guardians at the time of diagnosis.

Substantial research has been conducted on the transition to adult care for children with a variety of health conditions. In fact, recommendations on the inclusion of genetic counselors in the transition process have been released relating to individual genetic conditions such as Neurofibromatosis Type 1 and X‐Linked Hypophosphatemia (Borle et al., 2023; Dahir et al., 2022). However, there are limited studies describing best practices for transition for children with NDCs, and none specific to genetic counseling. This is concerning given that current studies suggest a genetic variant explaining an individual's NDC diagnosis can be identified in 15%–47.1% of patients (Murphy et al., 2016; Savatt & Myers, 2021). Given the recommendation by the American College of Medical Genetics for exome sequencing to be the first‐tier test for individuals with NDCs, genetic counselors are being integrated into the care team more frequently to assist in identifying an underlying genetic etiology (Manickam et al., 2021). GCs possess a knowledge of the etiology, medical implications and psychosocial sequela of a wide range of NDCs uniquely positioning them to aid in facilitating the transition process (Myers et al., 2020; Young‐Southward et al., 2017).

This study used a quantitative survey to explore GCs' current practices, attitudes, and perspectives on their potential role in discussing medical, social, and legal aspects of pediatric‐to‐adult care transition in patients with NDCs.

## 2 METHODS

The Indiana Institutional Review Board (IRB) deemed this study exempt (Protocol #19184). Informed consent was obtained from all eligible participants by selecting “*continue*” after reading the study information on the first page of the online survey.

### 2.1 Participants

An online survey was distributed through the National Society of Genetic Counselors (NSGC) and American Board of Genetic Counselors' (ABGC) research blast. Emails requesting distribution of the study information to their members were sent to two regional genetic counseling groups and 12 state genetic counseling organizations. Eligible participants were practicing GCs in the United States or Canada who currently provide genetic counseling for patients presenting with NDCs. The survey was open from May 24, 2023, until August 8, 2023. Approximately 4600 GCs received this survey invitation.

### 2.2 Instrumentation

This cross‐sectional study was carried out via an online survey designed in REDCap (Harris et al., 2009, 2019). Survey questions were independently developed and informed by two previous studies that explored the management of transition to adult healthcare (Geenen et al., 2003; Stepien et al., 2021).

The survey consisted of 58 questions including demographic information and GCs' awareness of institutional transition programs. Participants were asked how likely they were to discuss topics of transition at a post‐test counseling session with patients under 10 years, 10–12 years, 13–15 years, 16–18 years, and over 18 years of age. Additionally, one set of questions was developed for participants to answer twice: for patients aged 0–15 years and for those aged 16–22 years. These two age groups were chosen to determine if GCs' practices in discussing transition differed based on a patient age group that is younger, 0–15 years, compared to an age group in which transition from pediatric to adult care typically occurs, 16–22 years. Participants were asked about the frequency and likelihood of discussing transition and confidence in transition topics (on a 3‐point Likert scale, with 1 being ‘not at all confident’ and 3 being ‘very confident’). See Appendix S1 for the complete survey.

Participants were offered the option to enter a random drawing for one of five $50.00 Amazon gift cards by including their emails that were collected in a separate survey.

### 2.3 Data analysis

We utilized a Fisher's exact test to explore if the frequency of discussing topics of transition (never vs. at least once) was different for the two patient age groups (0–15 years vs. 16–22 years). To explore overall confidence, a non‐parametric Wilcoxon test was used to compare confidence scores between genetic counselors who indicated they wanted to play a role in transition compared to those who did not. Descriptive statistics are provided for all other variables.

## 3 RESULTS

### 3.1 Participants

A total of 56 GCs participated in the survey, resulting in an estimated response rate of 1.2%. Due to more than 50% incomplete survey responses, five participants out of the 56 were excluded from data analysis, yielding a final sample of 51 (91.1%). See Table 1 for participant demographics.

**TABLE 1:** Demographics (*n* = 51).

Demographic	Number of participants (%)
Gender	
Female	47 (92.2)
Male	2 (3.9)
Other	2 (3.9)
Race	
White	48 (94.1)
Asian	3 (5.9)
Years experience with pediatric patients	
1–5 years	30 (58.8)
6–10 years	17 (33.3)
11–15 years	1 (2.0)
15+ years	3 (5.9)
Genetics specialty	
Medical genetics	39 (76.5)
Developmental genetics	4 (7.8)
Neurogenetics	2 (3.9)
Metabolic genetics	2 (3.9)
Other	2 (3.9)
Cancer genetics	1 (2.0)
Cardiovascular genetics	1 (2.0)
Work setting	
Academic hospital/medical facility	33 (66.0)
Non‐profit hospital/medical facility	12 (24.0)
Public hospital/medical facility	4 (8.0)
Telegenetics or consulting	1 (2.0)
NSGC region	
Region 4 (AR, IA, IL, IN, KS, MI, MN, MO, ND, NE, OH, OK, SD, WI, Ontario)	20 (39.2)
Region 2 (DC, DE, MD, NJ, NY, PA, VA, WV, PR, VI, Quebec)	9 (17.6)
Region 5 (AZ, CO, MT, NM, TX, UT, WY, Alberta, Manitoba, Sask.)	8 (15.7)
Region 6 (AK, CA, HI, ID, NV, OR, WA, British Columbia, Yukon)	8 (15.7)
Region 3 (AL, FL, GA, KY, LA, MS, NC, SC, TN)	4 (7.8)
Region 1 (CT, MA, ME, NH, RI, VT, CN, Maritime provinces)	2 (3.9)
Aware of institutional transition process	
Yes	22 (44.0)
No	16 (32.0)
Unsure	12 (24.0)

### 3.2 Current practices

Participants were asked about the frequency of seeing patients with NDCs following the initial genetics assessment; 33.0% (*n* = 17) saw patients in follow‐up either never or once, 27.5% (*n* = 14) saw patients yearly, 19.6% (*n* = 10) reported 2–5 times a year, and 19.6% (*n* = 10) selected other.

**FIGURE 1:**
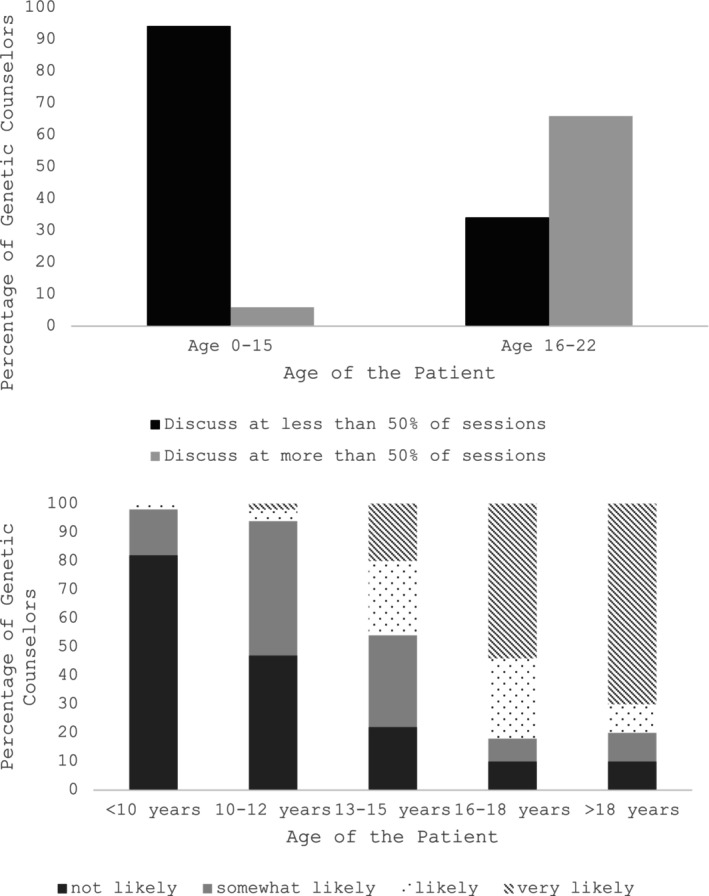
Genetic counselors' frequency of discussing topics of transition with patients depending on the age range at their appointment illustrated by age ranges 0‐15 years and 16‐22 years, and alternatively also broken down into age ranges <10 years, 10‐12 years, 13‐15 years, 16‐18 years, and >18 years to assess trends.

**FIGURE 2:**
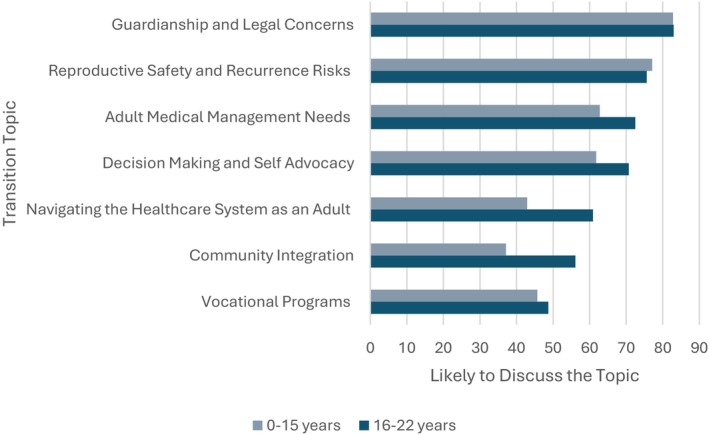
Percentage of genetic counselors who are likely/very likely to discuss seven different topics of transition with patients aged 0–15 years vs patients aged 16–22 years.

GCs reported being more likely to discuss the transition in over 50% of appointments with patients aged 16–22 years compared to patients aged 0–15 years (Fisher's exact *p* = 0.0003) (Figure 1). Age groups were further broken down into <10 years, 10–12 years, 13–15 years, 16–18 years, and 18+ years to assess trends. For patients under 10 years old, only 18% (*n* = 9) of genetic counselors were somewhat likely or likely to discuss topics of transition. With patients over 18 years, 70% of genetic counselors were very likely to discuss topics of transition. Figure 2 shows GCs' likelihood of discussing various topics of transition at a post‐test counseling appointment with patients presenting with NDCs at age 0–15 years, and for patients at the time of transition, age 16–22 years.

Participants who discussed topics of transition were asked about the importance of several factors in making the decision to discuss a particular topic. When asked about patients aged 0–15 years, 100% of participants indicated that questions asked by the patient or their guardian were a key factor, followed by complexity of future care (97.1%), and the presence of a NDC (65.7%). Similarly, at 16–22 years, 97.6% of participants responded that questions asked by the patient, or their guardians influenced their decision to discuss transition, complexity of future care by 95.1%, and presence of NDC was selected by 75.6%. Results describing factors impacting a decision not to discuss transition and barriers to discussion are provided in Tables S1 and S2, respectively.

### 3.3 Potential GC roles

**FIGURE 3:**
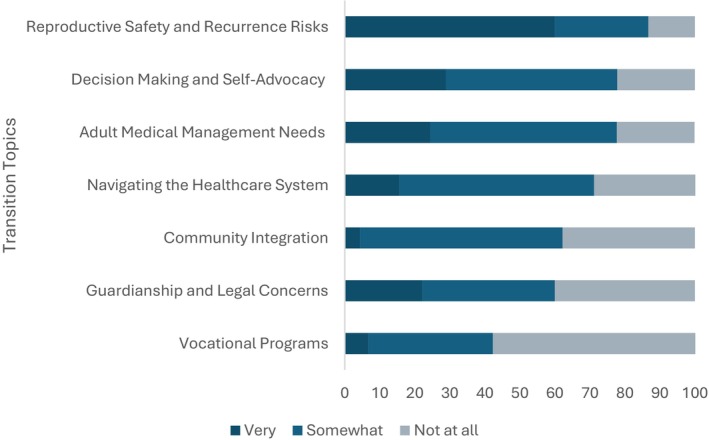
Genetic counselors' (*n* = 45) reported self‐confidence for discussing seven different topics of transition with pediatric patients presenting with neurodevelopmental conditions and their families.

All GCs perceived the transition planning process to be important (86.7%) or somewhat important (13.3%) for patients with NDCs. Over three‐quarters of respondents were interested in serving in the transition process (76.7%). The mean confidence score for the seven topics of transition was 1.91 on a scale of 1–3 (standard deviation = 0.48), Figure 3. Those interested in playing a role in transition had higher self‐confidence on average (mean confidence score = 2.03, standard error (SE) = 0.08) compared to those who were not interested in helping facilitate the transition process (mean score = 1.69, SE = 0.14, Wilcoxon *p* = 0.020).

## 4 DISCUSSION

Previous research has evaluated the practice of transition from pediatric to adult health care for patients from the perspectives of providers, caregivers, patients, and society (Geenen et al., 2003; Schrander‐Stumpel et al., 2007; Stepien et al., 2021). This study is the first to explore GCs' perspectives on their role in aiding in the transition of pediatric to adult care for children with NDCs. Our study was limited by the low response rate of 51 participants. The topic of transitional care is not something that has been frequently discussed in the genetic counseling profession which could have contributed to this low response rate. Those who did respond could have a bias toward an interest in transitional care.

### 4.1 Attitudes

Our results revealed all GCs who responded perceived transition planning for pediatric patients with NDCs as somewhat important or important, and more than three‐quarters expressed interest in participating in the transition process. We found confidence was positively associated with interest in being a part of the transition process. Unsurprisingly, topics for which most GCs indicated they felt most confident were discussing reproductive safety and recurrence risks. Participants reported being least confident in factors reflecting the social needs of a patient's diagnosis: vocational programs, community integration, and guardianship (Figure 3). Emphasis is placed on both the medical and social implications of genetic conditions within genetic counseling training practice‐based competencies (PBCs) (Doyle et al., 2016), however, our results suggest that confidence is not equal between the two domains of practice. This could be the result of limited or continuously changing government and community‐based resources dedicated to social support for individuals with NDCs.

### 4.2 Current practice

As a patient approaches the age at which transition to adult care occurs, GCs are more likely to discuss topics of transition. However, a retrospective analysis identified most children with NDCs underwent genetics evaluation between the ages of 3 and 5 years, and most GCs in our study only see a patient once for follow‐up (Du et al., 2022). Thus, patients with NDCs with an underlying genetic etiology may not interact with a GC as they approach the transitional care phase. In addition to patient age, most GCs noted questions asked by the patient/guardian were an important factor in deciding to discuss transition topics with patients in both age groups. While providing answers to questions is essential, not all families will inquire about care transition. Providing anticipatory guidance is key to empowering patients and families to navigate the diagnosis during all stages of life (Hyman et al., 2020).

## 5 CONCLUSIONS

Our results suggest GCs often do not discuss transitional care with patients with NDCs despite the perceived importance and desire to participate in the transition process. It is important for pediatric patients with NDCs to receive comprehensive transition care information pertaining to medical management and social integration. Genetic counselors have the necessary skills to communicate complex information to patients, which can be crucial for individuals with NDCs, and to empower them to be aware of their transitional needs. Given the limited follow‐up practices that were identified in our study, individuals with NDCs may have never spoken with a genetic counselor directly if they were evaluated as a child. These patients could benefit from evaluation with a genetic counselor to review their diagnosis in addition to receiving transitional care information. This study provides preliminary data on the attitudes and perspectives of GCs, as well as their potential role in transition discussions. Future research should examine the patient and family perspective on a desire to meet with a genetic counselor for an additional follow‐up near the age of transition. This would allow a discussion regarding the genetic diagnosis/additional genetic testing with the affected individual so that they might be more knowledgeable about their health and reproductive risks.

## AUTHOR CONTRIBUTIONS

Molly Lombard, Trisha Neidlinger, MS CGC, Leah Wetherill PhD, Jennifer Ivanovich, MS, CGC, and Deborah Hamby, MD contributed to the development of the project methodology and design. Molly Lombard was responsible for the recruitment of participants and data collection. Molly Lombard, Trisha Neidlinger, and Leah Wetherill confirm that they had full access to all the data in the study and take responsibility for the integrity of the data. Data analysis was completed by Leah Wetherill, and she takes responsibility for the accuracy of data analysis. This manuscript was initially drafted by Molly Lombard and all other authors contributed to the revision process. All authors gave final approval of this version to be published and agree to be accountable for all aspects of the work in ensuring that questions related to the accuracy or integrity of any part of the work are appropriately investigated and resolved.

## CONFLICT OF INTEREST STATEMENT

Molly Lombard, Leah Wetherill, PhD, Jennifer Ivanovich, MS CGC, Deborah Hamby, MD, and Trisha Neidlinger, MS CGC declare that they have no conflict of interest.

## ETHICS STATEMENT

Human studies and informed consent: This study was reviewed and granted an exemption by the Indiana University Institutional Review Board. All procedures followed were in accordance with the ethical standards of the responsible committee on human experimentation (institutional and national) and with the Helsinki Declaration of 1975, as revised in 2000. Implied informed consent was obtained for individuals who voluntarily completed the online survey and submitted their responses.

Animal studies: No non‐human animal studies were carried out by the authors for this article.

## Supporting information


Appendix S1

Appendix S2

## Data Availability

The data that support the findings of this study are available from the corresponding author upon reasonable request.
