# Supplementary material for: Exploring genetic counselors' interest and role in transitional care discussions for pediatric patients with neurodevelopmental conditions
Source: J Genet Couns. 2024 Nov 14;34(3):e1992. doi: 10.1002/jgc4.1992 (PMC12041836; doi:10.1002/jgc4.1992)
Supplement: Supplementary file 1 — Appendix S1 [file JGC4-34-0-s001.pdf]

Data Dictionary Codebook

Genetic Counselors and Transition (PID: 32362)

02/28/2024 1:21pm

Instruments

#	Variable / Field Name	Field Label *Field Note*	Field Attributes (Field Type, Validation, Choices, Calculations, etc.)
**Instrument: Study Information Sheet/Consent (study_information_sheetconsent) Enabled as survey**			
1	[study_id]	Study ID	text
2	[inclusion_criteria]	You are a genetic counselor that currently counsels pediatric patients presenting with neurodevelopmental delay.	yesno, Required<br>1 Yes<br>0 No
3	[inclusion_no]<br>Show the field ONLY if:<br>[inclusion_criteria] = '0'	This survey is designed for genetic counselors that currently counsel pediatric patients with an indication of neurodevelopmental delay. If you do not have any experience in this field we thank you for your time, but you do not meet the inclusion criteria.	descriptive
4	[study_information_sheetconsent_complete]	Section Header: *Form Status*<br>Complete?	dropdown<br>0 Incomplete<br>1 Unverified<br>2 Complete
**Instrument: Demographics (demographics) Enabled as survey**			
5	[gender]	What is your gender?	radio<br>1 Male<br>2 Female<br>3 Prefer not to answer<br>4 Other
6	[gender_other]<br>Show the field ONLY if:<br>[gender] = '4'	What is your gender?	text
7	[race]	Please select you race.	radio<br>1 White<br>2 Black or African American<br>3 American Indian or Alaska Native<br>4 Hispanic or Latino<br>5 Asian<br>6 Native Hawaiian or Other Pacific Islander<br>7 Other
8	[race_other]<br>Show the field ONLY if:<br>[race] = '7'	What is your race?	text

9	[years_practicing]	How many years have you been practicing as a genetic counselor?	radio<br>1 1-5 years<br>2 6-10 years<br>3 11-15 years<br>4 15+ years
10	[years_peds]	How many years experience do you have working with pediatric patients?	radio<br>1 1-5 years<br>2 6-10 years<br>3 11-15 years<br>4 15+ years
11	[clinics]	What genetic counseling clinics do you currently counsel pediatric patients with neurodevelopmental delay in?	radio<br>1 Cancer Genetics<br>2 Medical Genetics<br>3 Developmental Genetics<br>4 Neurogenetics<br>5 Cardiovascular Genetics<br>6 Metabolic Genetics<br>7 Multiple Clinics<br>8 Other
12	[clinics_other]<br>Show the field ONLY if:<br>[clinics] = '8'	What other clinics do you have experience with pediatric patients with neurodevelopmental delay in?	text
13	[primary_clinic]<br>Show the field ONLY if:<br>[clinics] = '7'	Which clinic do you see the most pediatric patients with neurodevelopmental delay in?	radio<br>1 Cancer Genetics<br>2 Medical Genetics<br>3 Developmental Genetics<br>4 Neurogenetics<br>5 Cardiovascular Genetics<br>6 Metabolic Genetics<br>7 Other
14	[primary_clinic_other]<br>Show the field ONLY if:<br>[primary_clinic] = '7'	Which clinic do you see the most patients with neurodevelopmental delay in?	text
15	[follow_up]	On average, how many times do you see a patient with neurodevelopmental delay following a diagnosis?	radio<br>1 Never<br>2 Once<br>3 2-5 Times<br>4 Yearly<br>5 Other
16	[follow_up_other]	On average, how many times do you see a patient with neurodevelopmental delay following a	text

	Show the field ONLY if: [follow_up] = '5'	diagnosis?	
17	[nsgc_region]	Please select the NSGC region in which you currently practice.	radio<br>1 Region 1: CT, MA, ME, NH, RI, VT, CN, maritime provinces<br>2 Region 2: DC, DE, MD, NJ, NY, PA, VA, WV, PR, VI, Quebec<br>3 Region 3: AL, FL, GA, KY, LA, MS, NC, SC, TN<br>4 Region 4: AR, IA, IL, IN, KS, MI, MN, MO, ND, NE, OH, OK, SD, WI, Ontario<br>5 Region 5: AZ, CO, MT, NM, TX, UT, WY, Alberta, Manitoba, Sask.<br>6 Region 6: AK, CA, HI, ID, NV, OR, WA, British Columbia, Yukon
18	[work_setting]	Please select your current employer's work setting.	radio<br>1 Academic Hospital/Medical facility<br>2 Public Hospital/Medical facility<br>3 Non-profit Hospital/Medical facility<br>4 Telegenetics or Consulting<br>5 Private company<br>6 Other
19	[work_setting_other]<br>Show the field ONLY if: [work_setting] = '6'	What is the setting of your current employer?	text
20	[institution_practice]	Are you aware of any practice within your department or institution that helps pediatric genetics patients with neurodevelopmental delay through the transition process?	radio<br>1 Yes<br>0 No<br>2 Unsure
21	[institution_model]<br>Show the field ONLY if: [institution_practice] = '1'	What type of practices or models are in place in your department/institution? Check all that apply.	radio<br>1 Designated transition facilitator assigned to patient<br>2 Genetics provider follows patient continuously through life<br>3 Multidisciplinary care conference or clinic<br>4 Detailed, written care plan<br>5 Separate transition program to refer patients<br>6 Other
22	[institution_model_other]<br>Show the field ONLY if: [institution_model] = '6'	Please describe the other practice in your department/institution.	text

23	[under_10]	Section Header: *How likely are you to discuss topics of transition at a follow-up counseling session if the patient is-*<br>Under 10 years	radio (Matrix)<br>1 Not likely<br>2 Somewhat likely<br>3 Likely<br>4 Very likely
24	[years_12]	10-12 years	radio (Matrix)<br>1 Not likely<br>2 Somewhat likely<br>3 Likely<br>4 Very likely
25	[years_15]	13-15 years	radio (Matrix)<br>1 Not likely<br>2 Somewhat likely<br>3 Likely<br>4 Very likely
26	[years_18]	16-18 years	radio (Matrix)<br>1 Not likely<br>2 Somewhat likely<br>3 Likely<br>4 Very likely
27	[over_18]	Over 18 years	radio (Matrix)<br>1 Not likely<br>2 Somewhat likely<br>3 Likely<br>4 Very likely
28	[demographics_complete]	Section Header: *Form Status*<br>Complete?	dropdown<br>0 Incomplete<br>1 Unverified<br>2 Complete
**Instrument: Follow-up Counseling Session (followup_counseling_session)** Enabled as survey			
29	[frequency_posttest]	How frequently do you discuss topics of transition at a follow-up counseling session with patients aged 0-15 years?	radio<br>1 Never (0% of the time)<br>2 Sometimes (up to 50% of the time)<br>3 Frequently (Over 50% of the time)<br>4 Always (100% of the time)
30	[ndd]<br>Show the field ONLY if: [frequency_posttest] = '2' or [frequency_posttest] = '3' or [frequency_posttest] = '4'	Section Header: *How important are each of the following factors in your decision to discuss topics of transition at a follow-up counseling session?*<br>Presence of neurodevelopmental delay	radio (Matrix)<br>1 Not important<br>2 Somewhat important<br>3 Important

			4 Very important
31	[care_management]<br>Show the field ONLY if: [frequency_posttest] = '2' or [frequency_posttest] = '3' or [frequency_posttest] = '4'	Complexity of future care management	radio (Matrix)<br>1 Not important<br>2 Somewhat important<br>3 Important<br>4 Very important
32	[questions]<br>Show the field ONLY if: [frequency_posttest] = '2' or [frequency_posttest] = '3' or [frequency_posttest] = '4'	Questions asked by patient or patient's guardians	radio (Matrix)<br>1 Not important<br>2 Somewhat important<br>3 Important<br>4 Very important
33	[navigating]<br>Show the field ONLY if: [frequency_posttest] = '2' or [frequency_posttest] = '3' or [frequency_posttest] = '4'	Section Header: *How likely are these topics to be included in your transition conversations?*<br>Navigating the health system as an adult	radio (Matrix)<br>1 Not likely<br>2 Somewhat likely<br>3 Likely<br>4 Very likely
34	[community]<br>Show the field ONLY if: [frequency_posttest] = '2' or [frequency_posttest] = '3' or [frequency_posttest] = '4'	Community Integration	radio (Matrix)<br>1 Not likely<br>2 Somewhat likely<br>3 Likely<br>4 Very likely<br>Field Annotation: ex. accessing community resources, making friends
35	[advocacy]<br>Show the field ONLY if: [frequency_posttest] = '2' or [frequency_posttest] = '3' or [frequency_posttest] = '4'	Decision making and self advocacy	radio (Matrix)<br>1 Not likely<br>2 Somewhat likely<br>3 Likely<br>4 Very likely<br>Field Annotation: ex. budgeting, mental health
36	[guardianship]<br>Show the field ONLY if: [frequency_posttest] = '2' or [frequency_posttest] = '3' or [frequency_posttest] = '4'	Guardianship and legal concerns	radio (Matrix)<br>1 Not likely<br>2 Somewhat likely<br>3 Likely<br>4 Very likely
37	[vocational]<br>Show the field ONLY if: [frequency_posttest] = '2' or [frequency_posttest] = '3' or [frequency_posttest] = '4'	Vocational programs	radio (Matrix)<br>1 Not likely<br>2 Somewhat likely<br>3 Likely

			4 Very likely
38	[repro_safety]<br>Show the field ONLY if: [frequency_posttest] = '2' or [frequency_posttest] = '3' or [frequency_posttest] = '4'	Reproductive safety and recurrence risks	radio (Matrix)<br>1 Not likely<br>2 Somewhat likely<br>3 Likely<br>4 Very likely
39	[adult]<br>Show the field ONLY if: [frequency_posttest] = '2' or [frequency_posttest] = '3' or [frequency_posttest] = '4'	Adult medical management needs	radio (Matrix)<br>1 Not likely<br>2 Somewhat likely<br>3 Likely<br>4 Very likely
40	[factors_no_posttest]<br>Show the field ONLY if: [frequency_posttest] = '1' or [frequency_posttest] = '2' or [frequency_posttest] = '3'	What factors impact your decision to not discuss topics of transition as a genetic counselor at a follow-up counseling session? Check all that apply.	checkbox<br>1 factors_no_posttest___1 Age of patient<br>2 factors_no_posttest___2 Lack of time in genetic counseling sessions<br>3 factors_no_posttest___3 Low self-confidence on discussing topics of transition<br>4 factors_no_posttest___4 Discussion lays outside of genetic counselor's scope of practice<br>5 factors_no_posttest___5 I do not often see patients at this age<br>6 factors_no_posttest___6 Other
41	[factors_no_posttest_other]<br>Show the field ONLY if: [factors_no_posttest(6)] = '1'	What other factors impact your decision to not discuss topics of transition at a post-test counseling session?	text
42	[followup_counseling_session_complete]	Section Header: *Form Status*<br>Complete?	dropdown<br>0 Incomplete<br>1 Unverified<br>2 Complete

Instrument: Time of Transition (time_of_transition) **Enabled as survey**

43	[frequency_transition]	How frequently do you discuss topics of transition at the time of transition? (Ages 16-22)	radio<br>1 Never (0% of the time)<br>2 Sometimes (up to 50% of the time)<br>3 Frequently (Over 50% of the time)<br>4 Always (100% of the time)
44	[ndd2]<br>Show the field ONLY if: [frequency_transition] = '2' or [frequency_transition] = '3' or [frequency_transition] = '4'	Section Header: *How important are each of the following factors in your decision to discuss topics of transition at the time of transition?*<br>Presence of neurodevelopmental delay	radio (Matrix)<br>1 Not important<br>2 Somewhat important<br>3 Important<br>4 Very important
45	[care_management2]<br>Show the field ONLY if: [frequency_transition] = '2' or [frequency_transition] = '3' or [frequency_transition] = '4'	Complexity of future care management	radio (Matrix)<br>1 Not important<br>2 Somewhat important<br>3 Important<br>4 Very important
46	[questions2]<br>Show the field ONLY if: [frequency_transition] = '2' or [frequency_transition] = '3' or [frequency_transition] = '4'	Questions asked by patient or patient's guardian	radio (Matrix)<br>1 Not important<br>2 Somewhat important<br>3 Important<br>4 Very important
47	[navigating2]<br>Show the field ONLY if: [frequency_transition] = '2' or [frequency_transition] = '3' or [frequency_transition] = '4'	Section Header: *How likely are these topics to be included in your transition conversations?*<br>Navigating the health system as an adult	radio (Matrix)<br>1 Not likely<br>2 Somewhat likely<br>3 Likely<br>4 Very likely
48	[community2]<br>Show the field ONLY if: [frequency_transition] = '2' or [frequency_transition] = '3' or [frequency_transition] = '4'	Community Integration	radio (Matrix)<br>1 Not likely<br>2 Somewhat likely<br>3 Likely<br>4 Very likely
49	[advocacy2]<br>Show the field ONLY if: [frequency_transition] = '2' or [frequency_transition] = '3' or [frequency_transition] = '4'	Decision making and self advocacy	radio (Matrix)<br>1 Not likely<br>2 Somewhat likely<br>3 Likely<br>4 Very likely
50	[guardianship2]<br>Show the field ONLY if: [frequency_transition] = '2' or [frequency_transition] = '3' or [frequency_transition] = '4'	Guardianship and legal concerns	radio (Matrix)<br>1 Not likely<br>2 Somewhat likely<br>3 Likely

			4 Very likely
51	[vocational2]<br>Show the field ONLY if: [frequency_transition] = '2' or [frequency_transition] = '3' or [frequency_transition] = '4'	Vocational programs	radio (Matrix)<br>1 Not likely<br>2 Somewhat likely<br>3 Likely<br>4 Very likely
52	[repro_safety2]<br>Show the field ONLY if: [frequency_transition] = '2' or [frequency_transition] = '3' or [frequency_transition] = '4'	Reproductive safety and recurrence risks	radio (Matrix)<br>1 Not likely<br>2 Somewhat likely<br>3 Likely<br>4 Very likely
53	[adult2]<br>Show the field ONLY if: [frequency_transition] = '2' or [frequency_transition] = '3' or [frequency_transition] = '4'	Adult medical management	radio (Matrix)<br>1 Not likely<br>2 Somewhat likely<br>3 Likely<br>4 Very likely
54	[factors_no_timetransition]<br>Show the field ONLY if: [frequency_transition] = '1' or [frequency_transition] = '2' or [frequency_transition] = '3'	What factors impact your decision to not discuss topics of transition as a genetic counselor at the time of transition?	checkbox<br>1 factors_no_timetransition___1 Age of the patient<br>2 factors_no_timetransition___2 Lack of time in genetic counseling sessions<br>3 factors_no_timetransition___3 Low self-confidence on discussing topics of transition<br>4 factors_no_timetransition___4 Discussion lays outside of genetic counselor's scope of practice<br>5 factors_no_timetransition___5 I do not often see patients at this age<br>6 factors_no_timetransition___6 Other
55	[factors_no_timetransition_other]<br>Show the field ONLY if: [factors_no_timetransition(6)] = '1'	What other factors impact your decision to not discuss topics of transition at a post-test counseling session?	text

56	[time_of_transition_complete]	Section Header: *Form Status*<br>Complete?	dropdown<br>0 Incomplete<br>1 Unverified<br>2 Complete
**Instrument: Future Practice (future_practice) Enabled as survey**			
57	[importance]	What is your perceived importance of transition planning for pediatric patients with neurodevelopmental delay?	radio<br>1 Not important<br>2 Somewhat important<br>3 Important
58	[interest]	Are you interested in playing a role in discussing topics of transition with your pediatric patients with neurodevelopmental delay?	yesno<br>1 Yes<br>0 No
59	[potential_roles]	Select all of the roles that you could see genetic counselors positively contributing to in a transition discussion.	checkbox<br>1 potential_roles___1 Navigating the health system as an adult<br>2 potential_roles___2 Community Integration<br>3 potential_roles___3 Decision making and self advocacy<br>4 potential_roles___4 Guardianship and legal concerns<br>5 potential_roles___5 Vocational programs<br>6 potential_roles___6 Reproductive safety and recurrence risks<br>7 potential_roles___7 Adult medical management needs<br>8 potential_roles___8 Other
60	[roles_other]<br>Show the field ONLY if:<br>[potential_roles(8)] = '1'	What other topics of transition do you feel a genetic counselor could positively contribute to?	text
61	[navigating_confidence]	Section Header: *Please report your confidence level on discussing the following transition topics?*<br>Navigating the health system as an adult	radio (Matrix)<br>1 Not at all confident<br>2 Somewhat confident<br>3 Very confident
62	[social_confidence]	Community Integration	radio (Matrix)<br>1 Not at all confident<br>2 Somewhat confident<br>3 Very confident
63	[decision_confidence]	Decision making and self advocacy	radio (Matrix)

			1 Not at all confident<br>2 Somewhat confident<br>3 Very confident
64	[guardianship_confidence]	Guardianship and legal concerns	radio (Matrix)<br>1 Not at all confident<br>2 Somewhat confident<br>3 Very confident
65	[vocation_confidence]	Vocational programs	radio (Matrix)<br>1 Not at all confident<br>2 Somewhat confident<br>3 Very confident
66	[repro_confidence]	Reproductive safety and recurrence risks	radio (Matrix)<br>1 Not at all confident<br>2 Somewhat confident<br>3 Very confident
67	[adult_confidence]	Adult medical management needs	radio (Matrix)<br>1 Not at all confident<br>2 Somewhat confident<br>3 Very confident
68	[logistical_barriers]	Please identify any logistical barriers that may inhibit you from potentially contributing to transition discussions with your patients?	checkbox<br>1 logistical_barriers___1 Lack of time in a ge[illegible] counseling session<br>2 logistical_barriers___2 Lack of genetic counselors in your department<br>3 logistical_barriers___3 Established pract[illegible] transition of pedia[illegible] patients in instituti[illegible]<br>4 logistical_barriers___4 Lack of training in t[illegible] topics<br>5 logistical_barriers___5 Limited resources<br>6 logistical_barriers___6 Patient needs/emotions/re[illegible]<br>7 logistical_barriers___7 Role assumed to be[illegible] by other provider[illegible]<br>8 logistical_barriers___8 Other
69	[barriers_other]<br>Show the field ONLY if:<br>[logistical_barriers(8)] = '1'	Please describe the other barriers.	text
70	[facilitator]	Section Header: *Please rank the following models in order of perceived effectiveness in regard to aiding pediatric patients with neurodevelopmental delay in the transition process. (1 being most effective and 5 being least effected)*	radio (Matrix - ranking)<br>1 1<br>2 2

		Designated transition facilitator assigned to patient at an institutional level	3 3<br>4 4<br>5 5
71	[followed_life]	Genetics provider follows patient continuously through life	radio (Matrix - ranking)<br>1 1<br>2 2<br>3 3<br>4 4<br>5 5
72	[care_conference]	Multidisciplinary care conference or clinic through life	radio (Matrix - ranking)<br>1 1<br>2 2<br>3 3<br>4 4<br>5 5
73	[care_plan]	Detailed, written care plan	radio (Matrix - ranking)<br>1 1<br>2 2<br>3 3<br>4 4<br>5 5
74	[program]	Separate transition program to refer patients associated with institution	radio (Matrix - ranking)<br>1 1<br>2 2<br>3 3<br>4 4<br>5 5
75	[model_1]	Please describe the benefits you see for the model you ranked #1 in the question above.	notes
76	[future_practice_complete]	Section Header: *Form Status*<br>Complete?	dropdown<br>0 Incomplete<br>1 Unverified<br>2 Complete
**Instrument: Incentive (incentive)** Enabled as survey			
77	[drawing]		descriptive<br>(Media URL: https://redcap.link/wx9p5yu6, Display format: Inline)
78	[incentive_complete]	Section Header: *Form Status*<br>Complete?	dropdown<br>0 Incomplete<br>1 Unverified

			2 Complete
