# Supplementary material for: Exploring genetic counselors' interest and role in transitional care discussions for pediatric patients with neurodevelopmental conditions
Source: J Genet Couns. 2024 Nov 14;34(3):e1992. doi: 10.1002/jgc4.1992 (PMC12041836; doi:10.1002/jgc4.1992)
Supplement: Supplementary file 2 — Appendix S2 [file JGC4-34-0-s002.docx]

| Supplemental Table 1. Barriers to Discussing Topics of Transition (n=44) | |
| --- | --- |
| **Barrier** | **Number of Participants (%)** |
| Lack of time in an appointment | 28 (63.6) |
| Lack of genetic counselors | 8 (18.2) |
| Established transition practice | 17 (38.6) |
| Lack of training | 27 (61.4) |
| Limited resources | 31 (70.5) |
| Patient needs | 15 (34.1) |
| Belief that this role is filled by another provider | 28 (63.6) |

| Supplemental Table 2. Factors Impacting the Decision to Not Discuss Topics of Transition | | | |
| --- | --- | --- | --- |
|  | **For Patients Aged 0-15 Years** | **For Patients Aged 16-22 Years** |  |
| **Factors** | **Number of Participants (%), n=47** | **Number of Participants (%), n=34** | **P-Value** |
| Age of a patient | 37 (78.7) | 14 (41.2) | 0.0055 |
| Lack of time in a genetic counseling appointment | 25 (53.2) | 19 (55.9) | 0.0001 |
| Low self-confidence on discussing transition | 13 (13.7) | 13 (38.2) | 0.0001 |
| Discussion lays outside of a GC's scope | 18 (38.3) | 17 (50.0) | 0.0001 |
| Not seeing patients at this age | 3 (6.4) | 4 (11.8) | 0.2214 |

^[[1]](#footnote-1)^

1. Utilized a Fisher’s exact test to explore if the five factors impacting a decision to not discuss transition were different for the two patient age groups (0-15 years vs 16-22 years) [↑](#footnote-ref-1)
